# Lung transplant referral practice patterns: a survey of cystic fibrosis physicians and general pulmonologists

**DOI:** 10.1186/s12890-020-1067-4

**Published:** 2020-03-04

**Authors:** Bethany L. Bartley, Carolyn E. Schwartz, Roland B. Stark, Anna M. Georgiopoulos, Deborah Friedman, Christopher J. Richards, Henry L. Dorkin, T. Bernard Kinane, Isabel P. Neuringer, Lael M. Yonker

**Affiliations:** 1 Department of Pediatrics, Division of Pulmonology, Massachusetts General Hospital, 55 Fruit Street, Boston, MA 02114 USA; 2 DeltaQuest Foundation, Inc., 31 Mitchell Road, Concord, MA 01742 USA; 3 Department of Psychiatry, Massachusetts General Hospital, 55 Fruit Street, Boston, MA 02114 USA; 4 Department of Medicine, Division of Pulmonology, Massachusetts General Hospital, 55 Fruit Street, Boston, MA 02114 USA; 5 Department of Pediatrics, Division of Pulmonology, Boston Children’s Hospital, Boston, MA 02115 USA

**Keywords:** Cystic fibrosis, Lung transplantation, Referral, Physician survey

## Abstract

**Background:**

Many individuals with cystic fibrosis (CF) die from respiratory failure without referral for lung transplant. Physician practices that may expedite, delay, or preclude referral, are poorly understood.

**Methods:**

Two parallel, web-based surveys focusing on lung transplant referral triggers and barriers, as well as pre-referral evaluation, were emailed to pulmonologists practicing in the New England region. One questionnaire was sent to CF providers (*n* = 61), and the second to general pulmonary providers practicing at the same institutions (*n* = 61).

**Results:**

There were 43 (70%) responses to the CF provider survey, and 25 (41%) responses to the general pulmonary (‘non-CF’) provider survey. Primary reasons for CF providers to refer their patients included: rapidly declining lung function (91%) and a forced expiratory volume in 1 s (FEV_1_) below 30% predicted (74%). The greatest barriers to referral for both CF and non-CF providers included active tobacco use (65 and 96%, respectively, would not refer), and active alcohol or other substance use or dependence (63 and 80%). Furthermore, up to 42% of CF providers would potentially delay their referral if triple-combination therapy or other promising new, disease-specific therapy were anticipated. In general, non-CF providers perform a more robust pre-referral medical work-up, while CF providers complete a psychosocial evaluation in higher numbers. Across both groups, communication with lung transplant programs was reported to be inadequate.

**Conclusions:**

Physician-level barriers to timely lung transplant referral exist and need to be addressed. Enhanced communication between lung transplant programs and pulmonary providers may reduce these barriers.

## Background

Lung transplant (LTx) is a potentially life-lengthening procedure for individuals with end-stage lung disease. To be considered a candidate, a patient’s longitudinal pulmonary provider typically makes a referral to a LTx center where the patient then undergoes an extensive medical and psychosocial evaluation. The International Society for Heart and Lung Transplantation (ISHLT) has published guidelines that recommend LTx referral for patients with a 2-year predicted survival of < 50% and a high likelihood of post-transplant survival [1]. The timing of referral is often more nuanced for pulmonologists, involving consideration of numerous patient- and disease-specific factors.

Advanced CF lung disease is the third most common indication for LTx in adults [2]. Disease-specific referral guidelines were first published by the CF Foundation (CFF) in 1998 [3] and revised in 2019 [4]. The original guidelines encouraged referral when a patient’s FEV_1_ reached < 30% predicted [3], as estimated two-year survival was < 50% for these individuals at that time [5]. Survival among this cohort has more than tripled in the interval between guidelines [6], however the updated guidelines now recommend early referral for adults with CF when their FEV_1_ is: (1) < 50% predicted and rapidly declining, (2) < 40% predicted with markers of shortened survival, or (3) < 30% predicted for all individuals [4].

Irrespective of lung function, additional factors may delay or even preclude a pulmonologist’s referral for LTx evaluation. A survey of CF center directors in 2015 identified physician knowledge regarding non-lung function–based recommendations for referral as a potential barrier to timely referral [7]. This regional survey of pulmonologists seeks to add to this literature by evaluating the potential influence of promising new disease-specific therapies (i.e. triple-combination CF transmembrane conductance regulator [CFTR] modulator therapies) and psychosocial factors including substance use and mental health history, on the timing of physician’s referral. Additionally, the level of work-up pulmonologists typically perform prior to referral and communication expectations from an accepting LTx program were evaluated. We hypothesized that referral patterns would differ between CF and general pulmonary (‘non-CF’) physicians and sought to identify potential areas of intervention to promote early LTx referral by CF providers.

## Methods

The study protocol was reviewed and approved by the Institutional Review Board of Partners HealthCare (Protocol #:2018P002262).

### Physician surveys and data collection

Two parallel online questionnaires were implemented in SurveyGizmo©; a 16-question survey of pediatric and adult pulmonologists specializing in CF care, and a 12-question survey of general adult pulmonologists who refer patients to LTx for disease indications other than CF (Additional files 1 and 2). Both questionnaires addressed factors that may influence referral timing, including physician practice setting and experience, as well as patient comorbidities, substance use, and age. Medical evaluation performed by the pulmonologist prior to referral and expectations for communication with LTx centers were also evaluated.

The CF provider questionnaire also contained questions assessing triggers for LTx referral and potential disease-specific precluding factors for referral building upon previously published national data [7]. The percentage of patients prescribed a CFTR modulator at the time of referral and the influence of CFTR-modulator therapy – either current or anticipated highly effective (i.e. triple-combination) therapies – on referral timing were also assessed. For comparison, the non-CF provider questionnaire evaluated the potential impact of a promising new, disease-specific therapy on referral timing.

The CFF-accredited care centers in the New England region of the United States host 10 pediatric, 9 adult, and 2 affiliate CF Care Programs. The CF-provider questionnaire was distributed via email to all of the CF pulmonologists practicing within this region (*n* = 61), and the parallel (non-CF) survey was sent to the same number of general pulmonologists practicing at the same institutions. Responses were collected over a 3-month interval from April to June 2019.

### Statistical analysis

Descriptive statistics were used to evaluate the frequency of responses to each survey item in the overall sample and by relevant subgroups. Statistical analyses were implemented using IBM SPSS Statistics for Windows, version 26 (IBM, Armonk, NY, USA).

## Results

There were 43/61 (70%) responses to the CF-provider survey, and 25/61 (41%) responses to the non-CF provider survey. Twenty-seven (63%) of the responding CF providers identified that they practice at pediatric CF programs. Of the non-CF pulmonary providers, all 25 reported that they primarily see adult patients (Table 1). The most common disease indication for LTx referral among non-CF providers was interstitial lung disease including idiopathic pulmonary fibrosis (*n* = 16, 64%), followed by chronic obstructive pulmonary disease (*n* = 4, 16%), and pulmonary vascular disease (*n* = 3, 12%). Two (8%) non-CF providers (e.g. pulmonologists not affiliated with a CFF-accredited center) identified CF as their most common disease indication for referral.

**Table 1:** Demographics of survey respondents, grouped by pulmonary provider type (e.g. cystic fibrosis (CF) or non-CF providers)

	All Respondents	CF Providers	Non-CF Providers
	*N* = 68	*N* = 43	*N* = 25
Hospital directly affiliated with a lung transplant center, *n* (%)	24 (35%)	13 (30%)	11 (44%)
Primary patient population, *n* (%)			
Pediatric	27 (40%)	27 (63%)	0
Adult	41 (60%)	16 (37%)	25 (100%)
Years independently practicing, *n* (%)^a^			
< 5 years	13 (19%)	7 (16%)	6 (24%)
5 to 15 years	28 (41%)	16 (37%)	12 (48%)
16 to 25 years	13 (19%)	9 (21%)	4 (16%)
> 25 years	13 (19%)	10 (23%)	3 (12%)
Number of patients referred to lung transplant annually by practice, *n* (%)^b^			
≤ 2 patients	27 (40%)	19 (44%)	8 (32%)
3–10 patients	35 (51%)	22 (51%)	13 (52%)
11–24 patients	1 (1%)	0 (0%)	1 (4%)
≥ 25 patients	4 (6%)	1 (2%)	3 (12%)

^a^One CF provider respondent preferred not to answer years in practice

^b^One CF provider respondent did not answer the number of patients referred to lung transplant annually

Of responding physicians (CF and non-CF combined), a majority (60%) had ≤15 years of practice experience (Table 1). The median number of patients referred for LTx annually by a physician’s practice was 3 and 5 individuals in the CF and non-CF provider groups, respectively. CF providers reported that ≥50% of patients referred to a LTx program in the prior year were prescribed a CFTR-modulator therapy prior to referral.

Responding CF providers primarily identified declining lung function as the primary trigger for referral; 91% would refer for a rapidly declining FEV_1_ and 74% would refer for a FEV_1_ < 30% predicted. A higher percentage of pediatric than adult CF pulmonologists indicated that a supplemental oxygen requirement, pulmonary hypertension, or refractory/recurrent pneumothorax would trigger their referral (Additional file 3: Figure S1). Referral threshold among CF providers with ≤15 years’ experience (*n* = 23) was lower than those with > 15 years’ experience (*n* = 19) with 83 vs. 63%, respectively, referring for an FEV_1_ < 30% predicted; 61 vs. 47% for increasing frequency of pulmonary exacerbations; 70 vs. 53% for recurrent hemoptysis; 74 vs. 53% for pulmonary hypertension, and 39 vs. 21% for decreased 6-min walk test (6-MWT) distance.

**Fig. 1:**
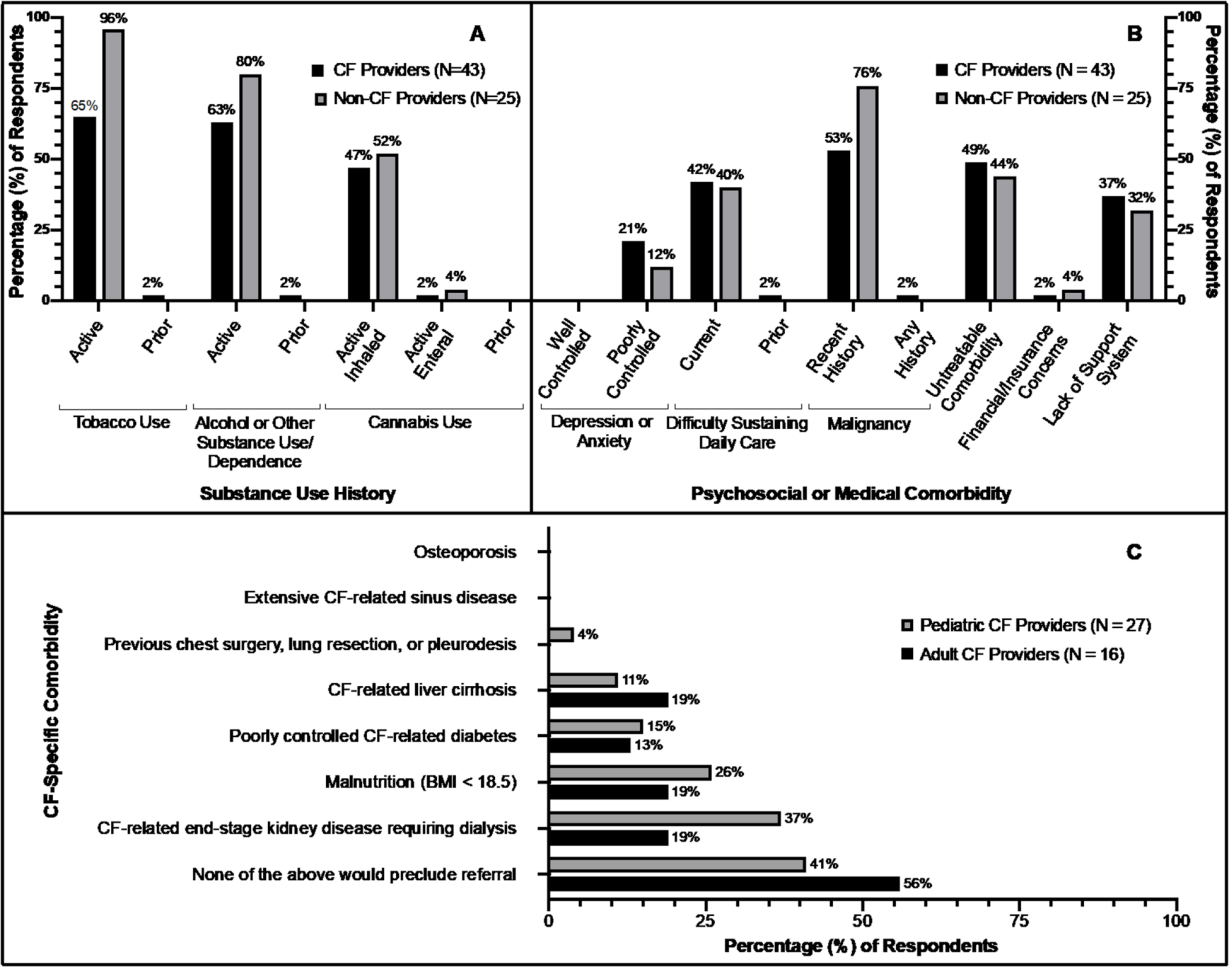
Absolute Contraindications: substance use history (**a**), psychosocial and medical comorbidities (**b**), and CF-specific comorbidities (**c**) that would preclude cystic fibrosis (CF) or non-CF pulmonary providers’ referral for lung transplant evaluation

Most CF and non-CF providers identified active substance use as precluding to referral (Fig. 1a). Specifically, 65% of CF providers and 96% of non-CF providers considered active tobacco use an absolute contraindication for referral. Sixty-three percent of CF providers and 80% of non-CF providers would preclude referral for active alcohol or substance use disorder, and 47% of CF providers and 52% of non-CF providers considered current inhaled cannabis use to be an absolute contraindication for referral. CF providers with more practice experience (> 15 years) were less likely to consider active substance use as an absolute contraindication to referral; only 58% would preclude their referral based on active tobacco, alcohol, or other substance use, and 32% would preclude their referral for current inhaled cannabis use. Few providers identified prior tobacco or other substance use with an extended period of sobriety as a contraindication for referral. Additionally, < 5% of providers in both groups identified current enteral cannabis use as an absolute contraindication.

Well-controlled depression or anxiety was notably not identified as a barrier to referral (Fig. 1b). However, 21% of CF providers and 12% of non-CF providers indicated that poorly controlled depression or anxiety despite supportive interventions and/or medications would preclude their referral. Similar to substance use, current difficulty sustaining adherence to daily medical therapy would preclude referral for many: 42% of CF providers and 40% of non-CF providers. However, a prior history of prolonged episodes of poor adherence to medical therapy would preclude < 5% of providers’ referral.

Many CF and non-CF providers identified malignancy within the past 2 years, and untreatable significant dysfunction of another major organ system as barriers to referral (Fig. 1b). CF-related end-stage kidney disease requiring dialysis and malnutrition (body mass index [BMI] < 18.5 kg/m^2^), were identified as the most common CF-related comorbidities that would preclude referral among CF providers (Fig. 1c). CF providers with ≤15 years of experience were more likely to preclude referral for poorly controlled CF-related diabetes, with 22% identifying this as an absolute contraindication, compared to only 5% of CF providers with > 15 years’ experience.

The potential influence of patient age and current or anticipated disease-specific therapy were also assessed. Both adult and pediatric pulmonologists were asked if pediatric age (≤18 years) would influence the timing of their referral. A third of CF providers and two-thirds of non-CF providers deferred this question (due to practice or preference) but a majority of the responding CF (24/29) and non-CF (5/9) pulmonologists said that pediatric age would not influence the timing of their referral; 2 non-CF providers would potentially delay their referral for pediatric age and the remaining minority of both groups would potentially expedite referral.

**Fig. 2:**
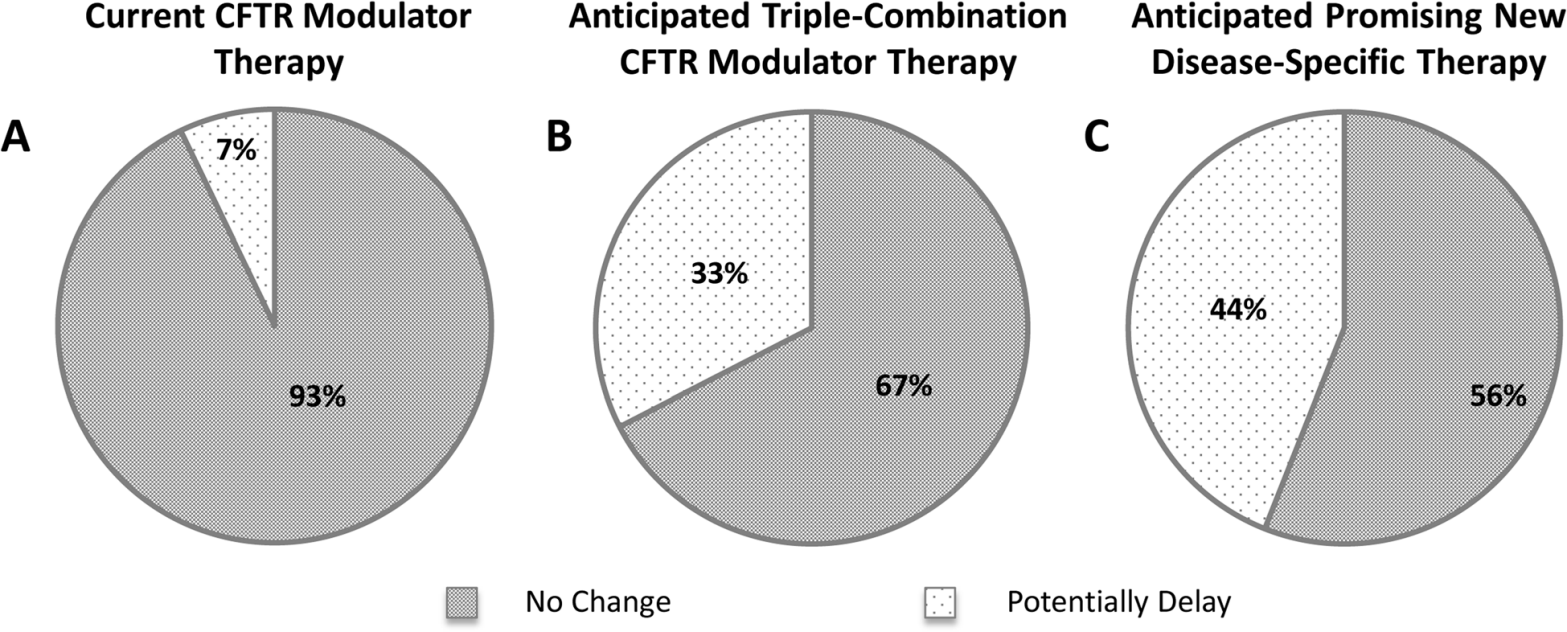
Referral Influencers: the potential impact of (**a**) current CFTR-modulator therapy, and (**b**) anticipated therapy with a highly effective CFTR modulator (i.e. triple-combination CFTR modulator or other promising new therapy) on the timing of CF providers’ referral for lung transplant evaluation, and the potential impact of (**c**) an anticipated promising new therapy targeting underlying disease process on the referral timing of non-CF pulmonary providers. Panels show the proportion of responding providers who would not change or potentially delay the timing of their referral based on these factors

Only 7% of CF providers indicated that they would potentially delay referral for patients currently taking a CFTR-modulator (Fig. 2a); however, 33% would delay referral for patients anticipated to soon qualify for a highly effective, CFTR-modulator therapy (Fig. 2b). Stratified by years of practice, CF providers with > 15 years’ experience were more likely to delay referral for anticipated triple combination CFTR-modulator therapy; 42% would potentially delay referral compared to 26% of CF providers with ≤15 years’ experience. Similarly, 44% of non-CF providers indicated that they would potentially delay their referral if they anticipated that their patient would soon qualify for a promising new disease-specific therapy (Fig. 2c).

Seventy-two percent of CF providers indicated that no specific colonizing organism would preclude their referral. Potential organisms that would preclude some provider’s referral included: *Burkholderia cepacia* complex (any) (7%), *Burkholderia multivorans* (2%), *Burkholderia cenocepacia* (23%), *Burkholderia dolosa* (16%), *Burkholderia gladioli* (2%), *Mycobacterium abscessus* (14%), and pan-resistant organisms (5%).

**Fig. 3:**
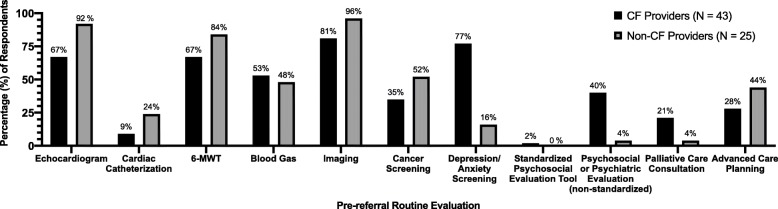
Medical and psychosocial evaluation routinely performed by pulmonary providers prior to lung transplant referral. Responses are grouped by cystic fibrosis (CF) and non-CF pulmonary providers

The degree of testing routinely performed by the pulmonologists prior to referral was also examined. A majority of both CF and non-CF providers stated that they routinely obtain an echocardiogram (67 and 92%, respectively), 6-MWT/physical therapy evaluation (67 and 84%), and chest imaging (81 and 96%) (Fig. 3). Non-CF providers obtain a cardiac catheterization and perform age- and gender-specific cancer screening in much higher numbers than their CF provider colleagues (24 and 52%, respectively, vs. 9 and 35%). Conversely, over three quarters of CF providers routinely perform depression or anxiety screening prior to referral, compared to 16% of non-CF providers. CF providers also more commonly perform a psychosocial or psychiatric evaluation prior to referral (40% vs. 4%) and obtain palliative care consultation in higher numbers (21% vs. 4%). Non-CF providers more commonly initiate advanced care planning discussions prior to referral (44% vs. 28%).

**Fig. 4:**
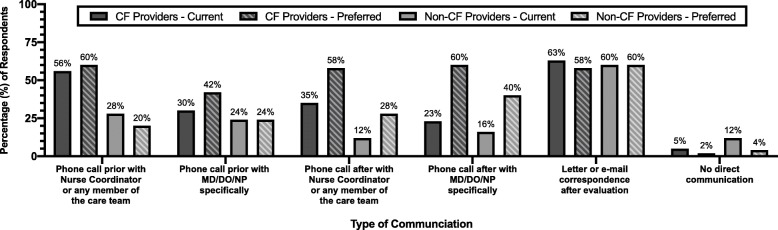
Types of communication typically occurring between lung transplant centers and referring pulmonary providers and types of communication identified as preferred among referring pulmonary providers. Responses are grouped by cystic fibrosis (CF) and non-CF pulmonary providers

Finally, providers where asked what types of communication they typically have with their referring LTx center currently, and their preferred mode(s) of communication peri-evaluation. A majority of CF and non-CF providers identified that they typically receive a letter or e-mail correspondence after the evaluation, which closely matches their preferred frequency of this communication (Fig. 4). Sixty percent of CF providers and 40% of non-CF providers indicated that they would prefer to receive a phone call after the evaluation from a LTx physician or nurse practitioner specifically, however only 23% of CF providers and 16% of non-CF providers stated that they receive this type of communication currently.

## Discussion

Lung transplant is a viable, potential life-extending treatment option with a proven survival benefit for individuals with CF and advanced lung disease [8]. Yet, many individuals with CF die each year without referral for LTx evaluation [6, 9]. Here we describe an in-depth analysis of the LTx referral process for pulmonary physicians, comparing CF and non-CF provider-referral patterns. This study adds to current research on physician-level referral barriers in CF [7] by evaluating the potential impact of substance misuse, psychosocial or medical comorbidity, patient age, and promising new therapies on provider referral, and furthermore identifies areas for improvement in pre-referral evaluation by CF providers, and communication expectations of lung transplant programs by their referring pulmonary providers.

### Barriers to referral

A high percentage of both CF and non-CF pulmonologists identified current tobacco use, alcohol dependence, and substance use as precluding patient-specific factors for referral consistent with ISHLT guidelines which consider these items to be absolute contraindications for LTx [1]. The updated CFF referral guidelines identify substance use as a modifiable barrier to transplant that should not preclude referral if persistent when a patient otherwise meets criteria [4]. This apparent contrast between recommendations highlights the need for pulmonologists to distinguish referral criteria from listing criteria for transplant; and not to defer referral based on the latter. With regards to substance use, ISHLT guidelines generally require abstinence and long-term participation in therapy before LTx is offered [1], hence referring early and considering dual referral to both a LTx program and substance misuse treatment program may more aptly allow time for substance misuse concerns to be addressed and ameliorated.

Poorly controlled depression or anxiety was also identified as a potential barrier to referral for some CF and non-CF pulmonologists in our study. CFF and European CF Society guidelines recommend annual screening for depression and anxiety in individuals with CF starting at age 12 [10, 11], which is possibly why markedly higher rates of pre-referral screening were observed among CF providers in this study. Treatment algorithms for managing depression and anxiety in CF Centers are available [10], and referral for mental health services may be particularly beneficial to improve symptoms in those with advanced disease [12]. Accordingly, the updated CFF referral guidelines also consider mental health concerns as modifiable barriers to transplant which need not be fully resolved prior to referral [4]. Our study demonstrates that this is not the current practice for over 20% of CF providers, indicating the need for ongoing mental health support in CF and consideration of early referral to LTx to familiarize patients with this team and partner in therapeutic management.

Many pulmonologists also indicated that poor medication adherence is a barrier to referral which again may reflect this item being considered an absolute contraindication for transplant by the ISHLT [1]. Pre-transplant non-adherence increases risk for non-adherence and mortality post-surgically [13], however perfect adherence to a complex CF treatment regimen consumes nearly 2 h each day [14], making adherence challenging for individuals with CF. Studies on CF patient-level barriers to LTx are limited but past non-adherence has been linked to feelings of inferior worthiness for transplant by patients [15]. Suitably, adherence behaviors are identified as a modifiable barrier to transplant in the updated CFF guidelines [4], reiterating the importance of early referral to identify and rectify this concern with reinforcement from the LTx team.

Some microorganisms pose a risk post-transplant [16], however we found that markedly fewer CF providers from our survey would preclude their referral for colonization with a specific organism (28% regionally vs. 68% nationally [7]). This regional discrepancy may reflect varied absolute contraindications among LTx programs, thus highlighting the updated CFF recommendations to consult local and geographically distant LTx centers for individuals with higher-risk organisms [4].

### Factors influencing referral timing

With regards to pediatric age patients, the updated CFF guidelines now recommend referral for children with CF (< 18 years) at a higher FEV_1_ threshold than adults due to their reduced survival given the same FEV_1_%-predicted [4, 17]. Despite improvement in pediatric survival post-LTx over the last few decades, adolescent recipients (age 11–17 years) continue to demonstrate the poorest survival among age groups with a median survival of 5.4 years post-transplant [18]. This may be why a small number of non-CF providers indicated that they would potentially delay their referral for patients < 18 years of age. However, the majority of pulmonologists (both CF and non-CF) indicated that pediatric age would not affect timing of referral, and many would expedite their referral for a patient < 18 years of age. Although LTx is far less common in the pediatric age group [2, 18], CF remains the most common indication for patients age 6 to 17 years [18], making monitoring of pediatric LTx referral patterns critical in guideline recommendations.

One of the most interesting results of this study is the potential negative impact of triple combination CFTR modulators (or other novel therapies) on provider referral. This is a hopeful era in CF care with improving life expectancy [19] and highly effective CFTR modulator therapies becoming available for nearly 90% of patients [20]. However, a third of pulmonologists specializing in CF and nearly half of the non-CF pulmonologists surveyed would potentially delay their referral for LTx evaluation if they anticipated their patient would soon qualify for a promising new, disease-specific therapy. These results unveil a potential bias against LTx as a treatment option for advanced lung disease, possibly related to perceptions of relative survival benefit. Although individuals with CF demonstrate superior post-transplant survival (median 9.9 years) compared to all other indications [2], pulmonologists may anticipate greater life-extending potential from new therapies despite their inherent lack of survival data.

### Pre-referral evaluation

Prior to referral, non-CF providers performed a more extensive medical evaluation, including echocardiogram and 6-MWT, while CF providers performed a more extensive psychosocial evaluation. Echocardiogram, venous blood gas, and 6-MWT are now recommended to screen for markers of disease severity in individuals with CF and FEV_1_ < 40% predicted (adults) or FEV_1_ < 50% predicted (pediatrics), prompting subsequent referral if indicated [4]. New resources such as the CFF Lung Transplant Referral Form [21] will hopefully encourage CF care teams to perform additional elements of pre-referral evaluation and potentially identify markers of shortened survival sooner.

### Communication expectations

Communication between CF providers and LTx centers is emphasized in the updated guidelines [4], and our study demonstrates that direct communication (e.g. phone call) expectations pre- and post-evaluation are not currently being met. Adding communication expectations to standardized LTx referral documentation may improve this parameter and enhance coordination of patient care. There are no specific recommendations for type of communication in the updated CFF guidelines; however, it is advised that CF and LTx care teams exchange information about transplant candidates at least every 6 months and with major clinical changes [4]. This study suggests that both CF and non-CF providers would prefer this contact to be a phone call.

### Limitations

The primary limitations of our physician survey include its relatively small sample size, lack of pediatric non-CF providers, and regional cohort of physicians, whose survey responses may not be generalizable to the practice patterns of providers on a national or international scale. Though notably in contrast to national CF provider survey data [7], this survey assesses the practice patterns of CF pulmonologists collectively, as opposed to program or center directors alone, offering a more complete assessment of LTx referral across the age spectrum in CF and provider experience level.

## Conclusions

The data yielded from our physician survey is highly relevant to the implementation of the new CFF consensus guidelines [4] which encourage early referral of individuals with CF to LTx programs. The impetus for this change from ‘timely’ to ‘early’ referral is in part due to poor predictive models for survival in advanced CF [6, 22], but primarily to allow individuals to be “medically, psychosocially, and financially prepared for LTx should the need arise” [4], and furthermore, to allow time to address modifiable barriers to LTx such as substance misuse and mental health concerns. Our study suggests that many CF provider’s current practice patterns differ from the updated recommendations, underscoring the need to educate providers on these new guidelines and their rationale.

## Supplementary information

**Additional file 1.** Cystic Fibrosis (CF) Provider Questionnaire.

**Additional file 2.** General Pulmonologist (Non-CF Provider) Questionnaire.

**Additional file 3: Figure S1.** Referral Triggers: clinical scenarios that would typically trigger cystic fibrosis (CF) providers’ referral for lung transplant evaluation in an individual with CF and advanced lung disease. Responses are grouped by CF program type (e.g. pediatric or adult). Providers were allowed to select multiple clinical scenarios.

## Data Availability

The data generated and analyzed for this study is available from the corresponding author on reasonable request.

## Abbreviations

6-MWT: 6-min walk test
BMI: Body mass index
CF: Cystic fibrosis
CFF: Cystic Fibrosis Foundation
CFTR: Cystic fibrosis transmembrane conductance regulator
FEV_1_: Forced expiratory volume in 1 s
ISHLT: International Society for Heart and Lung Transplantation
LTx: Lung transplant
