# Supplementary material for: Lung transplant referral practice patterns: a survey of cystic fibrosis physicians and general pulmonologists
Source: BMC Pulm Med. 2020 Mar 4;20:58. doi: 10.1186/s12890-020-1067-4 (PMC7055110; doi:10.1186/s12890-020-1067-4)
Supplement: Supplementary file 1 — Additional file 1. Cystic Fibrosis (CF) Provider Questionnaire. [file 12890_2020_1067_MOESM1_ESM.docx]

**Methods: Additional File 1**

Lung transplant referral practice patterns: a survey of cystic fibrosis and general pulmonologists

Bethany L. Bartley, MD

Carolyn E. Schwartz, ScD

Roland B. Stark, MEd

Anna M. Georgiopoulos, MD

Deborah Friedman, PhD

Christopher J. Richards, MD

Henry L. Dorkin, MD

T. Bernard Kinane, MD

Isabel P. Neuringer, MD

Lael M. Yonker, MD

**Cystic Fibrosis (CF) Provider Questionnaire:**

1. Choose your practice setting:
   1. CF program directly affiliated with a lung transplant program (Massachusetts General Hospital/Brigham and Women’s Hospital/Boston Children’s Hospital)
   2. CF program not directly affiliated with a lung transplant program (referral program)
   3. Prefer not to answer/Do not know
2. Choose your CF program type:
   1. Pediatric
   2. Adult
   3. Affiliate
   4. Prefer not to answer/Do not know
3. How many years since completing pulmonary fellowship have you been providing CF care?
   1. <5 years
   2. 5 to 15 years
   3. 16 to 25 years
   4. > 25 years
   5. Prefer not to answer/Do not know
4. Approximately how many patients with CF are referred for lung transplant evaluation from your program annually? *Please note this is the estimated number referred from your individual program (i.e. adult, pediatric, or affiliate), not the total for your CF Center which may be comprised of a pediatric, adult, and/or affiliate program(s).*  (fill in)
5. Approximately what percentage of patients with CF referred for lung transplant evaluation from your program in the past year were currently on a CFTR-modulator therapy? (Drop down box menu:100%, 90%, 80%, 70%, 60%, 50%, 40%, 30%, 20%, 10%, 0%, Prefer not to answer/Do not know)
6. Which of the following clinical scenarios would typically trigger your referral for lung transplant evaluation in a patient with CF and advanced lung disease? (check all that apply)
   1. FEV_1_ < 30% predicted
   2. FEV_1_ < 40% predicted
   3. A rapidly declining FEV_1_ despite optimal therapy
   4. Increasing frequency of pulmonary exacerbations requiring intravenous antibiotics
   5. Pulmonary exacerbation requiring ICU admission and/or non-invasive ventilation
   6. Refractory or recurrent pneumothorax
   7. Recurrent hemoptysis despite embolization procedure(s)
   8. Pulmonary hypertension
   9. Supplemental oxygen requirement
   10. Decreased 6-minute walk distance
   11. Prefer not to answer/Do not know
7. How would the timing of your referral for lung transplant evaluation change if a patient with CF were currently taking a CFTR-modulator therapy**?**
   1. It would not influence the timing of my referral
   2. It would potentially delay the timing of my referral
   3. It would potentially expedite the timing of my referral
   4. Prefer not to answer/Do not know
8. How would the timing of your referral for lung transplant evaluation change if you anticipated that your patient would soon qualify for a highly effective CFTR modulator therapy (i.e. triple-combination CFTR modulator or other promising new therapy)?
   1. It would not influence the timing of my referral
   2. It would potentially delay the timing of my referral
   3. It would potentially expedite the timing of my referral
   4. Prefer not to answer/Do not know
9. How would the timing of your referral for lung transplant evaluation change if a patient with CF were under 18 years of age?
   1. It would not influence the timing of my referral
   2. It would potentially delay the timing of my referral
   3. It would potentially expedite the timing of my referral
   4. I don’t routinely care for patients <18 years of age
   5. Prefer not to answer/Do not know
10. Colonization with which of the following potential organism(s) do you consider to be an absolute contraindication for lung transplant and if present, would preclude your referral for lung transplant evaluation in a patient with CF? (check all that apply)
    1. *Burkholderia cepacia* complex (any)
    2. *Burkholderia multivorans*
    3. *Burkholderia cenocepacia*
    4. *Burkholderia dolosa*
    5. *Burkholderia gladioli*
    6. *Mycobacterium abscessus*
    7. *Mycobacterium avium* complex
    8. *Aspergillus fumigatus* or history of allergic bronchopulmonary aspergillosis (ABPA)
    9. *Scedosporium* species
    10. Pan-resistant organisms (i.e. *Pseudomonas aeruginosa*, *Staphylococcus aureus, Stenotrophomonas maltophilia, Burkholderia cepacia* complex)
    11. None of the above colonizing organisms would preclude my referral for lung transplant evaluation
    12. Prefer not to answer/Do not know
11. Which of the following potential comorbidities or scenarios do you consider to be an absolute contraindication for lung transplant and if present, would preclude your referral for lung transplant evaluation in a patient with CF? (check all that apply)
    1. Recent history of malignancy, within the past 2 years
    2. Any history of malignancy
    3. Untreatable significant dysfunction of another major organ system (e.g. kidney, liver, heart, brain)
    4. Pulmonary hypertension
    5. Depression or anxiety, well controlled with supportive interventions and/or medication
    6. Depression or anxiety, poorly controlled despite supportive interventions and/or medication
    7. Lack of a reliable social support system
    8. Current difficulty sustaining daily care (e.g. poor adherence to medical therapy)
    9. Any history of prolonged episodes of poor adherence to medical therapy, despite current adherence
    10. Financial or insurance concerns
    11. None of the above items would preclude my referral for lung transplant evaluation
    12. Prefer not to answer/Do not know
12. Which of the following potential substance use histories do you consider to be an absolute contraindication for lung transplant and if present, would preclude your referral for lung transplant evaluation in a patient with CF? (check all that apply)
    1. Active alcohol or other substance use disorder (e.g., abuse/dependence)
    2. History of alcohol or other substance use disorder (e.g. abuse/dependence), now with an extended period of sobriety
    3. Active tobacco use
    4. History of tobacco use, quit > 6 months prior to the time of referral
    5. Current inhaled cannabis use
    6. Current enteral cannabis use
    7. History of cannabis use, not currently using
    8. None of the above would preclude my referral for lung transplant evaluation
    9. Prefer not to answer/Do not know
13. Which of the following CF-related comorbidities do you consider to be an absolute contraindication for lung transplant and if present, would preclude your referral for lung transplant evaluation in a patient with CF? (check all that apply)
    1. CF-related liver cirrhosis
    2. CF-related end-stage kidney disease requiring dialysis
    3. Poorly controlled CF-related diabetes
    4. Extensive CF-related sinus disease
    5. Osteoporosis
    6. Previous chest surgery, lung resection, or pleurodesis
    7. Malnutrition (BMI < 18.5)
    8. None of the above would preclude my referral for lung transplant evaluation
    9. Prefer not to answer/Do not know
14. Prior to referral for lung transplant evaluation, which of the following items does your CF program routinely perform, order, or recommend for patients with CF in which lung transplant referral is under consideration? (check all that apply)
    1. Echocardiogram
    2. Cardiac catheterization
    3. 6-minute walk test/physical therapy (PT) evaluation
    4. Venous or arterial blood gas
    5. Imaging (i.e. chest x-ray, chest CT scan)
    6. Age and gender specific cancer screening (i.e. colonoscopy, mammogram)
    7. Depression (PHQ-9) and anxiety (GAD-7) screening
    8. Screening with Stanford Integrated Psychosocial Assessment for Transplant (SIPAT), Psychosocial Assessment of Candidates for Transplantation (PACT), or other standardized psychosocial evaluation tool
    9. Psychosocial or psychiatric evaluation, not specifically using a standardized tool
    10. Palliative care consultation
    11. Advanced care planning
    12. None of the above
    13. Prefer not to answer/Do not know
15. At present, what type(s) of communication do you typically have with the lung transplant program that you most often refer your patients with CF to? (check all that apply)
    1. Phone call *prior* to the evaluation with a lung transplant RN Coordinator or any member of the care team
    2. Phone call *prior* to the evaluation with a lung transplant MD/DO/NP specifically
    3. Phone call *after* the evaluation with a lung transplant RN Coordinator or any member of the care team
    4. Phone call *after* the evaluation with a lung transplant MD/DO/NP specifically
    5. Letter or e-mail correspondence after evaluation
    6. No direct communication
    7. Prefer not to answer/Do not know
16. What type(s) of communication would you prefer with the lung transplant program that you refer your patients with CF to? (check all that apply)
    1. Phone call *prior* to the evaluation with a lung transplant RN Coordinator or any member of the care team
    2. Phone call *prior* to the evaluation with a lung transplant MD/DO/NP specifically
    3. Phone call *after* the evaluation with a lung transplant RN Coordinator or any member of the care team
    4. Phone call *after* the evaluation with a lung transplant MD/DO/NP specifically
    5. Letter or e-mail correspondence after evaluation
    6. No direct communication needed, expect the lung transplant team to ensure follow-up and good communication with the patient
    7. Prefer not to answer/Do not know

Comments (optional)

Please share below any additional comments regarding your CF center’s experience with the lung transplant referral process.

(Free Text)
