# Supplementary material for: Lung transplant referral practice patterns: a survey of cystic fibrosis physicians and general pulmonologists
Source: BMC Pulm Med. 2020 Mar 4;20:58. doi: 10.1186/s12890-020-1067-4 (PMC7055110; doi:10.1186/s12890-020-1067-4)
Supplement: Supplementary file 2 — Additional file 2. General Pulmonologist (Non-CF Provider) Questionnaire. [file 12890_2020_1067_MOESM2_ESM.docx]

**Methods: Additional File 2**

Lung transplant referral practice patterns: a survey of cystic fibrosis and general pulmonologists

Bethany L. Bartley, MD

Carolyn E. Schwartz, ScD

Roland B. Stark, MEd

Anna M. Georgiopoulos, MD

Deborah Friedman, PhD

Christopher J. Richards, MD

Henry L. Dorkin, MD

T. Bernard Kinane, MD

Isabel P. Neuringer, MD

Lael M. Yonker, MD

**General Pulmonologist (Non-CF Provider) Questionnaire:**

1. Choose your practice setting:
   1. Hospital directly affiliated with a lung transplant program (Massachusetts General Hospital/Brigham and Women’s Hospital/Boston Children’s Hospital)
   2. Hospital not directly affiliated with a lung transplant program (referral program)
   3. Prefer not to answer/Do not know
2. Choose your patient population:
   1. Primarily pediatric patients
   2. Primarily adult patients
   3. Mix of pediatric and adult patients
   4. Prefer not to answer/Do not know
3. How many years since completing pulmonary fellowship have you been providing pulmonary care?
   1. <5 years
   2. 5 to 15 years
   3. 16 to 25 years
   4. > 25 years
   5. Prefer not to answer/Do not know
4. Approximately how many patients are referred for lung transplant evaluation from your practice annually? (fill in)
5. Which of the following is the most common disease indication for lung transplant referral from your practice?
   1. Interstitial lung disease, including idiopathic pulmonary fibrosis (IPF)
   2. Cystic fibrosis
   3. Chronic obstructive pulmonary disease (COPD), including emphysema
   4. Pulmonary vascular disease
   5. Other (fill in)
   6. Prefer not to answer/Do not know
6. How would the timing of your referral for lung transplant evaluation change if you anticipated that your patient would soon qualify for a promising new therapy targeting their underlying disease process?
   1. It would not influence the timing of my referral
   2. It would potentially delay the timing of my referral
   3. It would potentially expedite the timing of my referral
   4. Prefer not to answer/Do not know
7. How would the timing of your referral for lung transplant evaluation change if your patient were under 18 years of age?
   1. It would not influence the timing of my referral
   2. It would potentially delay the timing of my referral
   3. It would potentially expedite the timing of my referral
   4. I don’t routinely care for patients <18 years of age
   5. Prefer not to answer/Do not know
8. Which of the following potential comorbidities or scenarios do you consider to be an absolute contraindication for lung transplant and if present, would preclude your referral for lung transplant evaluation in a patient? (check all that apply)
   1. Recent history of malignancy, within the past 2 years
   2. Any history of malignancy
   3. Untreatable significant dysfunction of another major organ system (e.g. kidney, liver, heart, brain)
   4. Pulmonary hypertension
   5. Depression or anxiety, well controlled with supportive interventions and/or medication
   6. Depression or anxiety, poorly controlled despite supportive interventions and/or medication
   7. Lack of a reliable social support system
   8. Current difficulty sustaining daily care (e.g. poor adherence to medical therapy)
   9. Any history of prolonged episodes of poor adherence to medical therapy, despite current adherence
   10. Financial or insurance concerns
   11. None of the above items would preclude my referral for lung transplant evaluation
   12. Prefer not to answer/Do not know
9. Which of the following potential substance use histories do you consider to be an absolute contraindication for lung transplant and if present, would preclude your referral for lung transplant evaluation in a patient? (check all that apply)
   1. Active alcohol or other substance use disorder (e.g., abuse/dependence)
   2. History of alcohol or other substance use disorder (e.g. abuse/dependence), now with an extended period of sobriety
   3. Active tobacco use
   4. History of tobacco use, quit > 6 months prior to the time of referral
   5. Current inhaled cannabis use
   6. Current enteral cannabis use
   7. History of cannabis use, not currently using
   8. None of the above would preclude my referral for lung transplant evaluation
   9. Prefer not to answer/Do not know
10. Prior to referral for lung transplant evaluation, which of the following items does your practice routinely perform, order, or recommend for patients in which lung transplant referral is under consideration? (check all that apply)
    1. Echocardiogram
    2. Cardiac catheterization
    3. 6-minute walk test/physical therapy (PT) evaluation
    4. Venous or arterial blood gas
    5. Imaging (i.e. chest x-ray, CT scan)
    6. Age and gender specific cancer screening (i.e. colonoscopy, mammogram)
    7. Depression (PHQ-9) and anxiety (GAD-7) screening
    8. Screening with Stanford Integrated Psychosocial Assessment for Transplant (SIPAT), Psychosocial Assessment of Candidates for Transplantation (PACT), or other standardized psychosocial evaluation tool
    9. Psychosocial or psychiatric evaluation, not specifically using a standardized tool
    10. Palliative care consultation
    11. Advanced care planning
    12. None of the above
    13. Prefer not to answer/Do not know
11. At present, what type(s) of communication do you typically have with the lung transplant program that you most often refer your patients to? (check all that apply)
    1. Phone call *prior* to the evaluation with a lung transplant RN Coordinator or any member of the care team
    2. Phone call *prior* to the evaluation with a lung transplant MD/DO/NP specifically
    3. Phone call *after* the evaluation with a lung transplant RN Coordinator or any member of the care team
    4. Phone call *after* the evaluation with a lung transplant MD/DO/NP specifically
    5. Letter or e-mail correspondence after evaluation
    6. No direct communication
    7. Prefer not to answer/Do not know
12. What type(s) of communication would you prefer with the lung transplant program that you refer your patients to? (check all that apply)
    1. Phone call *prior* to the evaluation with a lung transplant RN Coordinator or any member of the care team
    2. Phone call *prior* to the evaluation with a lung transplant MD/DO/NP specifically
    3. Phone call *after* the evaluation with a lung transplant RN Coordinator or any member of the care team
    4. Phone call *after* the evaluation with a lung transplant MD/DO/NP specifically
    5. Letter or e-mail correspondence after evaluation
    6. No direct communication needed, expect the lung transplant team to ensure follow-up and good communication with the patient
    7. Prefer not to answer/Do not know

Comments (optional)

Please share below any additional comments regarding you or your practice’s experience with the lung transplant referral process.

(Free Text)
