# Supplementary material for: Lung transplant referral practice patterns: a survey of cystic fibrosis physicians and general pulmonologists
Source: BMC Pulm Med. 2020 Mar 4;20:58. doi: 10.1186/s12890-020-1067-4 (PMC7055110; doi:10.1186/s12890-020-1067-4)
Supplement: Supplementary file 3 — Additional file 3: Figure S1. Referral Triggers: clinical scenarios that would typically trigger cystic fibrosis (CF) providers’ referral for lung transplant evaluation in an individual with CF and advanced lung disease. Responses are grouped by CF program type (e.g. pediatric or adult). Providers were allowed to select multiple clinical scenarios. [file 12890_2020_1067_MOESM3_ESM.docx]

**Results: Supplementary Figure**

Lung transplant referral practice patterns: a survey of cystic fibrosis and general pulmonologists

**Figure S1. Referral Triggers:** clinical scenarios that would typically trigger cystic fibrosis (CF) providers’ referral for lung transplant evaluation in an individual with CF and advanced lung disease. Responses are grouped by CF program type (e.g. pediatric or adult). Providers were allowed to select multiple clinical scenarios.
